# Monitoring Weaning Stress in Fillies and Colts on a Thoroughbred Breeding Farm by Cortisol and Blood Inflammatory Markers: The Benefits of Gradual Separation and Social Support

**DOI:** 10.3390/ani15243551

**Published:** 2025-12-10

**Authors:** Ömer Deniz, Hüseyin Serkan Erol, René van den Hoven, Ali Cesur Onmaz, Francesca Aragona, Francesco Fazio

**Affiliations:** 1 Department of Internal Medicine, Faculty of Veterinary Medicine, Kastamonu University, Kastamonu 37150, Türkiye; 2 Department of Biochemistry Balıkesir Türkiye, Faculty of Veterinary Medicine, Balıkesir University, Balıkesir 10100, Türkiye; 3 Department of Companion Animals and Horses, Equine University Clinic for Internal Medicine and Infectious Diseases, Veterinary University of Vienna, Veterinärplatz 1, A-1210 Vienna, Austria; prof.renevandenhoven@gmail.com; 4 Department of Internal Medicine, Faculty of Veterinary Medicine, Erciyes University, Kayseri 38280, Türkiye; 5 Department of Veterinary Science, University of Messina, Via Giovanni Palatucci, 98168 Messina, Italy

**Keywords:** foal, weaning, stress, cytokine, inflammatory biomarkers, cortisol

## Abstract

Weaning represents a major developmental milestone for foals, especially when 4- to 7-month-old foals are abruptly separated from their dams. Studies have shown that the post-weaning period is associated with physiological and psychological stress, which may affect immune function. The present study aimed to describe and analyze the pattern of blood inflammatory biomarkers related to the innate immune system. A gradual foal–dam separation procedure was chosen, which included post-weaning social buffering by a familiar adult horse. Twelve Thoroughbred foals (six fillies, six colts) aged 4–6 months were enrolled and divided into a colt and a filly group, and after weaning, were kept as such. Blood samples were collected before and up to 7 days after weaning. Serum cortisol and cytokines (IL-2, IL-6, IL-10, TNF-α, IFN-γ) were quantified using equine-specific ELISA kits. Routine descriptive statistics were used to present the data, and inferences were drawn using a two-way repeated measures ANOVA. The level of significance was set at *p* < 0.05. Results showed significant differences in the concentrations of IL-10 (*p* < 0.001), IFN-γ (*p* < 0.01), and TNF-α (*p* < 0.01) on various days post-weaning. An apparent significant sex effect was not shown, but IL-10 peaked on day 3 in fillies. IFN-γ decreased significantly by day 5 in both sexes. Conversely, TNF-α levels increased significantly by day 5. No significant changes were observed for cortisol.

## Simple Summary

Weaning is a critical but stressful event in a foal’s life. If not adequately managed, welfare is threatened, and health may be compromised. This study describes daily changes in inflammatory biomarkers in blood in response to a weaning protocol after gradual separation from the dam and post-weaning in a colt and a filly group alongside familiar adult horses. A convenience sample of a group of six Thoroughbred fillies and six colts, all aged 4–6 months, was monitored. Blood samples were collected on the day before the definitive separation and over the next 7 days. Cortisol and cytokines (IL-2, IL-6, IL-10, TNF-α, IFN-γ) were measured on various days. Results showed some significant changes between pre- and post-weaning samples for IL-10, IFN-γ, and TNF-α. There were no statistically significant sex-related differences calculated for any of the parameters. Importantly, cortisol levels remained stable, suggesting that following the protocol could reduce weaning stress. Overall, while cytokine shifts reflected the physiological challenge, the absence of heightened cortisol indicated a blunted stress response. This gradual and socially supportive approach presents a practical, evidence-based method for safeguarding foal welfare, supporting healthy development, and enhancing resilience. Moreover, the use of inflammatory biomarkers could be a useful indicator of the physiological response to weaning in foals.

## 1. Introduction

Weaning represents a critical developmental milestone in mammals, marking the transition from maternal milk dependence to nutritional autonomy. The timing and physiological consequences of weaning vary widely among species and are shaped by evolutionary trade-offs between maternal investment, offspring growth, and future reproductive success [1,2].

Horses are precocial mammals, such as most ungulates. They are born with advanced motor and thermoregulatory capabilities, stand and suckle within hours after birth, and achieve nutritional independence at an earlier developmental stage [3].

An excellent summary of weaning in free ranging horses and under domestic conditions is given by Henry et al. (2020) [4]. Briefly, in feral horses, weaning takes place gradually over several months through the joint initiative of the dam and the foal. An increase in maternal rejection at the end of lactation is an important step in the weaning process, which finally results in weaning at the age of 9–11 months, sometimes even shortly before the birth of the next foal. In contrast, most breeders of horses kept in hobby farms or studs traditionally separate their foals abruptly between the age of 4 to 7 months [4]. The separation from the dam is not just a nutritional shift but also causes significant emotional and physiological challenges [3]. The impact on wellbeing was recently clearly made plausible by the study of Delank et al. 2023 [5].

Hoffman et al. (1995) used serum cortisol as a suitable marker of stress in their study of weaning stress in terms of behavioral and biochemical observations during an ACTH challenge [6].

Concurrent with hypercortisolaemia, the inflammatory cytokine profiles in blood show documented changes during weaning in various mammalian species. Studied potential markers include pro-inflammatory cytokines such as IL-1β, IL-6, and TNF-α and could indicate the activation of the innate immunity [7,8,9].

The objective of the present study was to describe the effect of weaning on foals after 2 weeks of gradual separation from their dams on their blood cortisol, anti-inflammatory and pro-inflammatory cytokines.

## 2. Materials and Methods

The present experimental protocol was approved by the Kastamonu University Animal Experiments Local Ethics Committee (Decision no: 2024/37). The study was conducted at the regional Golkoy Breeding Farm in Kastamonu, Turkey (Latitude: 41.371; Longitude: 33.7756; Altitude: 800.0 m). Informed consent was obtained from the owners of the foals before the start of the study. Twelve Thoroughbred foals (6 fillies, 6 colts) were enrolled in the experimental trial.

Foals were aged between 4 and 6 months, with a body weight ranging from 200 to 260 kg. The animals included in the study were all used for the weaning process; control groups were not available. All animals were clinically monitored by evaluating physiological parameters, including rectal temperature, respiratory rate, and heart rate, by experienced farm staff and a veterinarian with at least 10 years of experience in veterinary care. Physiological clinical examination fell within the physiological ranges for the equines [10]. Foals were fed alfalfa hay twice a day, to which they had become accustomed for 2 weeks before the weaning procedure. For the entire protocol, the foals were kept in the same environmental and management conditions. Colts and fillies were kept in two separate groups. Each group was in contact with the other adult horses. After weaning, the foals were fed with good-quality alfalfa hay. On the first day after weaning, 0.5 kg of a concentrate was fed, and the quantity was gradually increased to 1 kg twice daily by day 5. With this scheme, the risk of overeating by dominant foals was reduced. The concentrate was supplemented with 0.175 mycotoxin binder (Toxifix) and a vitamin–mineral premix (Kavimix). The cereal composition of the concentrate was: 63.5% oats, 23.33% barley, 8.33% extracted soybean–corn blend, 4.17% wheat bran. The salt content was 0.5%. During the same period, a gastroprotective (Gastrogard (0.9 mg/lb (2 mg/kg)) and a probiotic supplement (Yea-Sacc) were administered. Water was also available ad libitum.

### 2.1. Protocol

Each foal was handled daily by the same trained operator (a farm staff member experienced in foal management) to reduce stress and ensure consistent human contact. The interaction time between the foal and the operator was gradually increased from approximately 5 min on the first day to 1 h by the end of the observation period.

To minimize stress, the weaning protocol began before the definitive separation from the dams. During this period (14 days of gradual separation), the foals were accustomed by their operators to being visually separated from their dams by gradually increasing the time spent with the operator (1 h per day). After 14 days, the foals, all familiar with each other, were weaned from their dams and split into filly (n = 6) and colt (n = 6) groups. These sex groups were established and populated with adult barren mares and geldings that were already accustomed to socialization, to maintain a peaceful hierarchy.

### 2.2. Sampling and Laboratory Analysis

Jugular venous blood samples were taken on the day before weaning (T0), one day after weaning (T1), 3, 5, and 7 days after weaning (T3, T5, and T7, respectively). The blood samplings were performed at 13:00 to minimize variance, especially with respect to feeding-induced, possibly dominant effects on the physiological daily oscillation of inflammatory cytokines, as has been shown to occur in horses [11].

No blood samples were taken during the two-week gradual separation step. The degree of distress foals experienced during this phase of the study was not precisely evaluated using a behavioral evaluation protocol. The sampling time points were carefully chosen to monitor both the most obvious changes in stress and immune responses [12].

Blood samples were centrifuged at 3000 rpm for 10 min to obtain serum, which was aliquoted and immediately stored at −80 °C until analysis. Serum concentrations of cortisol, interleukins 2, 6, 10, interferon gamma (IFN-γ) and tumor necrosis factor-α (TNF-α) were assessed using enzyme-linked immunosorbent assay kits specific for equine species (Horse Cortisol COR ELISA Kit, sensitivity 0.25 ng/mL, Lbiont (Zhangjiang Town, Pudong New Area, Shanghai, China), Equine IL-2 ELISA kit, sensitivity 0.22 ng/mL, Lbiont; Equine IL-6 ELISA kit, sensitivity 0.52 ng/L, Lbiont, IL-10 ELISA kit, sensitivity 0.08 ng/L, Lbiont, TNF-α ELISA kit, sensitivity 0.55 ng/L, Lbiont, IFN-γ ELISA kit, sensitivity 0.75 ng/mL). A micro-well plate reader (BioTek ELX800, BioTek, Winooski, VT, USA) was used to read the absorbance values of blank and standard samples at 450 nm after 15 min of incubation. The concentration of each analysis was determined from the resulting optical densities (OD) used to calculate each standard curve.

The intra-assay CV% was <8%, and inter-assay CV% was <10%. All analyses were performed in duplicate to ensure accuracy and reproducibility. The validity and sensitivity of each kit have been verified by the standard curves provided by the manufacturer.

### 2.3. Statistical Analysis

The normal distribution of the data was tested using the Kolmogorov–Smirnov test. The time and sex effects on all parameters were investigated using two-way ANOVA for repeated measures, if appropriate, followed by post hoc multiple comparison tests. Data were analyzed with statistical software GraphPad Prism v. 9.5.1 (GraphPad Software Ltd., Boston, MA, USA). Results were reported as means ± standard deviation (SD); a *p*-value < 0.05 was set for significance. An a priori sample size calculation was performed to determine the minimum number of horses to be sampled (G*Power 3.1 Software, Heinrich-Heine-Universität, Düsseldorf, Germany). Accepting an effect size (f) of 0.6, a significance level (α) of 0.05, and a power (1 − β) of 0.80, 12 horses were necessary to find statistically significant differences.

## 3. Results

Table 1 presents the mean results over time for cortisol and cytokine concentrations. The inflammatory cytokines showed a time-dependent effect on the concentrations of IL-10 (*p* < 0.001), IFN-γ (*p* < 0.01), and TNF-α (*p* < 0.01). There was no significant overall sex effect on the concentrations. However, fillies tended to have occasional peak IL-10 concentrations compared to colts at 3 and 5 days post-weaning (*p* < 0.01; *p* < 0.001). A significant decrease in IFN-γ was observed at T5 compared to T0 (*p* < 0.01). Additionally, compared to T0 at T5, there was a substantial increase in TNF-α (*p* < 0.01). No significant differences were observed for cortisol (*p* = 0.14, *p* = 0.92), IL-2 (*p* = 0.31, *p* = 0.63), and IL-6 (*p* = 0.34, *p* = 0.87).

## 4. Discussion

In the first week after weaning, mean blood cortisol concentrations remained unchanged compared to the day before weaning and continued to do so until the end of the week. Sex had no statistically significant effect on serum cortisol concentration, as confirmed by some previous weaning studies [6]. Apparently, the foals were not under stress. On the other hand, Delank et al. (2023) showed increased fecal cortisol metabolites [5,6,7,8,9,10,11,12,13] as a proxy for cortisol metabolism that indicated that foals had not fully acclimated to the new situation. However, whether this indicated stress could not be reliably associated with observed behavioral signs of stress or poor coping with separation from their dams. Hypercortisolaemia is generally assumed to be a marker of stress [14]; however, current insights suggest that hypercortisolaemia per se may not be directly caused by stress. Associations between cortisol and stress may be mediated by metabolic factors [15]. Anyhow, given that glucocorticosteroids have many other downstream effects [16], this could include the suppression of immune function. The latter is what we tried to show by monitoring the serum cytokine patterns in weaned foals.

Regarding the effect of weaning on markers of innate immunity, we observed that IFN-γ concentration decreased significantly after weaning, whereas TNF-α concentration increased. Adams and Horohov (2013) also found this for IFN-γ, but not for TNF-α, which was reduced in their study [17]. This may possibly have been caused by the abrupt weaning of the foals in their research. IFN-γ repeatedly emerges as a cytokine of interest across studies, suggesting that gradual weaning and stress mitigation strategies may reduce immune suppression. However, findings differ by methodology (ELISA vs. qPCR) and sample timing, making comparison difficult. Elevated post-weaning TNF-α levels indicate an inflammatory response.

Overall, the kinetics (rise and fall) of IL-6 and TNF-α, balanced by IL-10, offer the most informative, semi-quantitative representation of systemic innate immune activity. Despite the fact that cytokine production is compartmentalized and plasma concentrations are low and transient, changes in blood cytokine kinetics seem sufficiently sensitive, at least, to display widespread or systemic innate immune activation [18]. Hence, the temporal patterns can indicate a present increase in intensity of activation or regulatory failure in the immune response. Nevertheless, findings should be interpreted alongside hematological and blood biochemical parameters, such as leukocyte counts and acute-phase proteins, for a reliable assessment [19,20,21,22,23,24]. Consequently, temporal cytokine profiles, particularly the balance between IL-6, TNF-α, and IL-10, may offer a useful but indirect and semi-quantitative measure of innate immune status and should be interpreted in combination with cellular and clinical inflammatory markers [25,26,27].

We could not show a difference between the sexes for most of the immunologic parameters, except for the observed interdependence of IL-10 at D3 in fillies. There is some agreement that colts are more resilient to weaning-related stress compared to fillies [28,29].

In conclusion, our study findings support those of other studies that have shown gradual weaning to be less harmful to the wellbeing of foals compared to abrupt weaning.

The conceptual weakness of this study is the lack of behavioral observation supporting the assessment of the foal’s psychological status. The sampling time frame of 7 days should have been 21 days including the 14 days of gradual separation of foals from dams. Furthermore, only a small number of commonly used inflammatory markers were employed to characterize the principal remarkable complexity of this process. Potentially, at least 100 different cytokines or chemokines may be involved [30]. Although this was not obvious in our study, circulating IL-6 is considered the most reproducible systemic biomarker of inflammation and prognosis in sepsis, trauma, and severe viral disease, particularly when evaluated longitudinally rather than as a single measurement [31]. Furthermore, TNF-α and IL-10 contribute complementary information, as suggested by our study as well. Nevertheless, cytokine concentrations in plasma correlate only weakly with local tissue production, underscoring their role as better markers of systemic than of compartmentalized innate responses [32]. Studies combining multiplex cytokine panels with machine-learning models have shown enhanced predictive accuracy for disease severity and outcomes compared with single markers [33]; this approach may be used to inform future studies on aspects of foals’ innate immunity. Since advances in blood transcriptomics and proteomics enable broader assessment of innate immune status, these techniques warrant further consideration as they offer greater reproducibility than isolated cytokine values [34].

## 5. Conclusions

This study demonstrates that gradual weaning induces measurable but limited alterations in circulating cytokines in foals. Evidence of avoiding physiological stress responses was suggested by the cortisol concentrations, which remained stable throughout the observation period. Among the cytokines examined, IFN-γ decreased and TNF-α increased in the days following weaning, indicating a modest shift in innate immune activation. Other cytokines, including IL-2, IL-6, and IL-10, showed only minor or transient fluctuations, with no consistent differences between sexes except for slightly higher IL-10 levels in fillies at certain time points. We concluded that gradual weaning as conducted according to our weaning protocol is less disruptive to the immune and physiological status of foals than abrupt weaning. Future research using extended sampling periods, larger cytokine panels, and complementary clinical or transcriptomic markers may provide a more comprehensive understanding of innate immune dynamics during the weaning process.

## Figures and Tables

**Table 1:** Mean± Standard Deviation of all the inflammatory biomarkers with their unit of measurement in colts and fillies before weaning (T0) and after weaning (T1, T3, T5 and T7). * *p* < 0.05 vs. T0; ^A^ *p* < 0.05 vs. T3.

	Blood Parameters	Time				
		T0	T1	T3	T5	T7
Fillies	Cortisol ng/ml	37.1 ± 10.5	39.1 ± 9.5	38.5 ± 7.5	32.7 ± 9.2	33.0 ± 7.6
	IL-2 ng/L	5.3 ± 2.3	4.6 ± 1.8	5.3 ± 2.6	4.3 ± 2.1	4.0 ± 1.3
	IL-6 ng/L	5.5 ± 1.2	5.7 ± 0.7	5.4 ± 0.6	5.1 ± 0.7	5.3 ± 0.3
	IL-10 ng/L	3.4 ± 1.1	2.7 ± 0.4 ^A^	4.1 ± 1.6	2.3 ± 0.8 ^A^	2.6 ± 1.0
	IFN-γ ng/mL	11. ± 2.6	10.7 ± 1.6	10.4 ± 1.7	8.6 ± 2.7 *	9.1 ± 0.9
	TNF-α ng/mL	13.9 ± 1.2	16.1 ± 2.6	17.6 ± 4.6	17.6 ± 5.1 *	18.1 ± 4.2
Colts	Cortisol ng/ml	35.5 ± 4.9	36.2 ± 3.0	39.1 ± 5.9	39.3 ± 10.8	31.3 ± 4.1
	IL-2 ng/L	4.6 ± 1.1	4.8 ± 1.3	4.4 ± 0.6	5.5 ± 1.7	4.2 ± 0.4
	IL-6 ng/L	5.1 ± 0.4	5.0 ± 0.4	5.3 ± 0.2	5.3 ± 0.7	5.7 ± 1.7
	IL-10 ng/L	3.1 ± 0.4	2.7 ± 0.3	3.1 ± 0.3	3.3 ± 0.7	2.8 ± 0.2
	IFN-γ ng/mL	10.1 ± 0.3	9.6 ± 0.6	9.4 ± 0.5	8.0 ± 1.1 *	8.7 ± 0.6
	TNF-α ng/mL	12.9 ± 3.3	15.4 ± 0.6	18.2 ± 2.9	18.0 ± 4.9 *	18.6 ± 8.5

## Data Availability

The raw data supporting the conclusions of this article will be available from the authors on request.
